# Median ages at stages of sexual maturity and excess weight in school children

**DOI:** 10.1186/1742-4755-10-56

**Published:** 2013-10-19

**Authors:** Alexandre P Luciano, Jucemar Benedet, Luiz Carlos de Abreu, Vitor E Valenti, Fernando de Souza Almeida, Francisco AG de Vasconcelos, Fernando Adami

**Affiliations:** 1 Programa de Pós-Graduação em Ciências da Saúde e Departamento de Saúde da Coletividade, Faculdade de Medicina do ABC, Av. Príncipe de Gales, 821, Santo André, SP 09060-650, Brasil; 2 Laboratório de Escrita Científica, Faculdade de Medicina do ABC, Av. Príncipe de Gales, 821, Santo André, SP 09060-650, Brasil; 3 Programa de Pós-Graduação em Nutrição, Universidade Federal de Santa Catarina, Campus Universitário Reitor João David Ferreira Lima, Florianopolis, SC 88040-970, Brasil; 4 Departamento de Fonoaudiologia, Faculdade de Filosofia e Ciências, UNESP, Av. Hygino Muzzi Filho, 737, Marilia, SP 17525-900, Brasil; 5 Programa de Pós-Graduação em Educação Física, Universidade Federal de Santa Catarina, Campus Universitário Reitor João David Ferreira Lima, Florianopolis, SC 88040-970, Brasil

**Keywords:** Adolescents, Obesity, Early puberty, Scholar children

## Abstract

**Background:**

We aimed to estimate the median ages at specific stages of sexual maturity stratified by excess weight in boys and girls.

**Materials and method:**

This was a cross-sectional study made in 2007 in Florianopolis, Brazil, with 2,339 schoolchildren between 8 to 14 years of age (1,107 boys) selected at random in two steps (by region and type of school). The schoolchildren were divided into: i) those with excess weight and ii) those without excess weight, according to the WHO 2007 cut-off points for gender and age. Sexual maturity was self-evaluated by the subjects according to the Tanner sexual development stages, and utilizing median ages for the genitalia, breasts, and pubic hair stages.

**Results:**

In the boys with excess weight, precocity was observed in the stages 4 for genitals and pubic hair and 2 for pubic hair, with the values for excess and normal weight. The median ages at the beginning of puberty (stage 2–sexual development) for boys and girls in Florianopolis were 10.8 and 10.3 years, respectively.

**Conclusion:**

Excess weight is associated with lower median ages in the sexual maturity stages in boys and girls and that it should be taken into account when evaluating sexual maturity in children and adolescents.

## Background

Puberty is considered as a series of maturation events that are interrelated and promote body changes, such as the development of reproductive function, and the appearance of secondary sexual characteristics [1]. During the puberty period individuals develop sexual maturity, which consists of biological maturation, defined as a progression process towards a mature biological state and cellular differentiation [2,3]. The evaluation of sexual maturity is the most widely used method in population studies for evaluation of biological maturity and growth due to its easy application and low cost.

Through the evaluation of age at the onset of puberty, a syndrome called precocious sexual maturity, characterized by an accelerated maturation process, has been discovered. The main consequence of this syndrome is short adult stature when compared with a person within the range of normality [4].

The timing of puberty onset may be affected by the environmental factors. The status of energy stores is crucial in the control of pubertal onset and progression, which means that both situations of under and overnutrition ca influence timing of sexual maturation [5].

Obesity has presented itself as an important modifying variable, advancing sexual maturity [6]. Puberty is a crucial period in relation to the development of obesity in boys and girls. Various studies have investigated the relationship that exists between sexual maturity and obesity [7-9]. The literature uses sexual maturity stages and the menarche age as indicators of sexual maturity, with age as the indicator of precocity [5,10].

The determination of age at the puberty onset may be utilized as an indicator to monitor growth. It is recognized, however, that few studies have considered such a parameter, stratifying the analyses by excess weight and using representative samples. Therefore, we aimed to evaluate the estimation of the median ages at the sexual maturation stages stratified by excess weight in boys and girls.

## Method

This is a cross-sectional study performed in 2007, between May and September, in Florianópolis, SC, in the south of Brazil. The city presents elevated indices of social development and health indicators, with infant mortality rates of 8/1,000 live births and a Human Development Index (HDI) of 0.875, which are higher than the national averages of 19.5/1,000 and 0.813, respectively [11]. This study was approved by the Ethical Committee in Research (Number 028/06).

A probabilistic population sample of students from 7 to 14 years old from public and private schools of the city was taken. According to the Health Secretary of the city, the population of students in this age in 2006 was 53,679 (25,619 from 7 to 10 years old, and 28,060 from 11 to 14 years old). All the students from 7 to 14 years old, from public and private schools, and residing in Florianopolis were considered eligible.

To calculate the sample size, the following variables was considered: prevalence of excess weight of 22.1% [12]; margin of error of 2 percentage points; design effect of 1.3; level of significance of 5%, and power test of 80%. For losses, 10% was added, totaling, in the final calculation 3,100 schoolchildren from 7 to 14 years old. Excluding seven years-old children, whose data for sexual maturity was not collected, the final sample for this study consisted of 2,412 schoolchildren from 8 to 14 years old (with 1,144 boys, 47.4%).

The study used a two-step sampling process. The first step divided the schools into four strata according to geographical region (downtown or coastal) and type of school (public or private). In each stratum, schools were selected randomly: from a total of 87 schools (33 private and 54 public), 20% were selected (17, from which 11 were public and 6, private). In the second step, 30% of the children from each school were randomly chosen to compose the sample.

The anthropometric measurements were taken according to the protocol by the World Health Organization (WHO) [13], proposed by Lohman et al. [14] The body weight was measured using a Marte electronic scale, model PP 180, with capacity for 180 Kg and a 100 g scale; the stature was measured using an Alturaexata^®^ stadiometer with a 1 mm scale.

The team responsible for collecting the data was formed by 10 people previously trained in a workshop held on September 2006 and March 2007. The workshop consisted of theoretical and practical classes on anthropometric measurements, followed by a pilot-study in two schools (one public and the other private) which were excluded from selection for the later collection of data, where there was also a study to determine the intra and inter evaluator mistakes of the anthropometric measurements [15].

Excess weight was determined by the growth curves from the Body Mass Index (BMI) according to gender and age, proposed by the World Health Organization [16]. The students were divided into two groups: i) those with excess weight and ii) those without excess weight. The height and the BMI were also analyzed by the Z score values (stature Z score and BMI Z score) obtained by the LMS values according to age and gender, as explained below. The value of 5 was considered biologically plausible for stature and BMI Z scores [16].

Since BMI varies in childhood and adolescence according to age and gender, the z-score for the BMI variable had to be calculated. The new references for evaluation of the nutritional status of Brazilian children based on the distribution of BMI values [17] were used. To calculate the z-score, LMS values were used, by age and gender, according to the following formula:

$$Z\;\text{score}\;\text{BMI}=[{\left(\text{BMI}/M\right)}^{L}-1]/\left(\text{LS}\right)$$

The LMS sums up the data in smoothed curves that are specific to each stratum, which in this case are the ages and genders. Parameter M is the median value of the index observed inside each stratum; parameter S is the coefficient of variation for each stratum; and parameter L is the Box-Cox coefficient employed for the mathematical transformation of the values of the variable in question in order to obtain a normal distribution in each stratum [18,19].

Sexual maturity was evaluated according to the development stages proposed by Tanner [20], which consists of five developmental stages for genitals (boys), breasts (girls), and pubic hair (boys and girls). The procedure adopted was self-evaluation, wherein the subjects were given individual orientation and chose the stages they were by themselves. As there is no standard definition of early sexual maturity, we used an approach similar to other studies in order to ensure comparability of our results [21-25]. Premature sexual maturity definition was based on the decimal median age for each one of the five sexual maturity stages for each gender.

The weight of the schoolchildren at birth was provided by their parents or responsible, who were instructed to consult their health log books. The subjects were classified as underweight (<2,500 g), normal weight (≥2,500 g–3,999 g) or overweight (≥4,000 g) [26].

The nutritional state of the mother was evaluated by the BMI, with self-evaluated weight and stature. The classification of excess weight (≥25 kg/m) followed the recommendation of the World Health Organization [26].

The information on commuting to school was evaluated through a Previous Day Food Questionnaire (PDFQ-3), previously validated [27]. The commute to school was classified as active (walking or by bicycle) or non-active (by car, bus, or by a ride on a motorcycle or bicycle).

The students’ data (name, date of birth, school year, and type of school) was obtained from documents supplied by the school. The type of school was classified as public or private.

Due to the non-normality of anthropometric data (Shapiro-Wilk test, p < 0.05), the choice was made to describe the quantitative variables through the median and 25 and 75 percentiles; for the qualitative variables the relative frequency (%) was used. The comparison of the quantitative variables was made by the Mann–Whitney test (between genders) and Krustal-Wallis (between sexual maturity classification–premature, normal, late). The association between the qualitative variables was investigated by the Rao-Scott test. The comparison of the median ages at each sexual maturity stage according to excess weight and gender was made by the Mann–Whitney test. The statistical program used was the Stata 11.0, using the svy command analyses to ponder them according to sampled weights and strata [28,29].

## Results

Seventy-three individuals (3.03% of loss) were excluded for the following reasons: stature Z scores higher than 5 (one girl); missing or inconsistent data for sexual maturity (36 boys and 36 girls), resulting in a final number of 2,339 students from 8 to 14 years old (1,107 boys). No significant statistical differences in prevalence of excess weight, values of BMI Z score, stature Z score and other independent variables were found when comparing the excluded schoolchildren with those who composed the sample.

Boys represented 47.7% of the population of the study. When compared to the girls, they presented higher values of BMI Z score (p < 0.001), stature Z score (p = 0.002), prevalence of current excess weight (34.4 vs 24.0%, p < 0.001), and excess birth weight (14.4 vs 8.1%, p < 0.001), with no differences found in the other variables (Table 1).

**Table 1:** Characteristics of the boys and girls studied

**Study variables**	**Boys (47.7%)**	**Girls (52.3%)**	**p**^**§**^
	Median (p25 ; p75)*		
Age (years)	11 (10;13)	12 (10;13)	0.53
BMI Z score	0.51 (-0.22;1.42)	0.21 (-0.41;0.96)	<0.001
Stature Z score	0.25 (-0.41;0.99)	0.11 (-0.52;0.79)	0.002
	%		
Excess weight	34.4	24.0	<0.001
Sexual Maturity Classification			0.94
Precocious	33.1	32.5	
Late	32.2	32.7	
Mother’s excess weight	31.0	33.4	0.31
Weight at birth (grams)			<0.001
< 2,500	5.8	8.3	
≥ 4,000	14.4	8.1	
Active commute to school	43.6	45.0	0.56
Type of school			0.97
Public	65.4	65.5	
Private	34.6	34.5	

* p25 and p75; percentiles 25 and 75, respectively.

^§^ Mann–Whitney test (quantitative variables) and Rao-Scott test (qualitative variables).

Brazil, 2007.

Tables 2 and 3 show the median ages for sexual maturity stages in boys and girls stratified by excess weight. In boys with excess weight (Table 2) we see precocity in stage 4 genitalia and pubic hair, and in stage 2 pubic hair, with the values for excess and normal weights being, respectively: 13.3 vs 13.7 years for genitalia (stage 4); 13.3 vs 13.8 (stage 4-pubic hair); and 10.8 vs 11.3 (stage 2-pubic hair). In girls (Table 3), except for stage 5, all stages show median ages lower than individuals with excess weight, which is the same tendency observed for pubic hair.

**Table 2:** Median ages in sexual maturity stages of boys according to excess weight classification

**Stages**	**Genitalia**			**Pubic hair**		
	**Without excess weight**	**With excess weight**	**p**^**§**^	**Without excess weight**	**With excess weight**	**p**^**§**^
	Median (p25–p75)*			**Median (p25–p75)**		
	**Age (years)**			**Age (years)**		
1	8.8	8.6	0.55	8.9	8.7	0.32
	(7.8–10.2)	(7.6–9.8)		(7.8–10.2)	(7.8–9.8)	
2	10.8	10.5	0.27	11.3	10.8	0.008
	(9.5–12.0)	(9.3–11.7)		(10.0–12.2)	(9.5–11.8)	
3	12.6	12.3	0.06	12.7	12.3	0.36
	(11.9–13.5)	(11.3–13.3)		(11.9–13.4)	(11.5–13.4)	
4	13.7	13.3	0.01	13.8	13.3	<0.001
	(12.8–14.3)	(12.8–13.8)		(13.2–14.4)	(12.7–13.8)	
5	14.0	13.7	0.2	14.2	13.9	0.09
	(13.2–14.4)	(12.8–14.3)		(13.7–14.4)	(13.2–14.2)	

* p25 and p75: percentiles 25 and 75, respectively.

^§^ Mann–Whitney test. Brazil, 2007.

**Table 3:** Median ages in sexual maturity stages of girls according to excess weight classification

**Stages**	**Breasts**			**Pubic hair**		
	**Without excess weight**	**With excess weight**	**p**^**§**^	**Without excess weight**	**With excess weight**	**p**^**§**^
	Median (p25–p75)*			**Median (p25–p75)**		
	**Age (years)**			**Age (years)**		
1	8.7	7.9	0.00 2	8.8	8.3	0.00 6
	(7.7–9.7)	(7.5–8.8)		(7.8–9.8)	(7.6–9.2)	
2	10.5	9.7	<0.0 01	11.0	10.5	0.04
	(9.7–11.6)	(8.6–10.7)		(9.9–11.7)	(9.5–11.5)	
3	12.4	11.5	<0.0 01	12.4	12.3	0.06
	(11.8–13.4)	(10.7–12.5)		(11.8–13.4)	(11.3–12.9)	
4	13.7	13.0	<0.0 01	13.5	13.1	0.02
	(12.9–14.3)	(12.3–13.8)		(12.8–14.2)	(12.3–14.0)	
5	13.8	13.8	0.16	13.8	13.4	0.16
	(13.5–14.3)	(13.0–14.2)		(13.5–14.3)	(13.0–14.3)	

* p25 and p75: percentiles 25 and 75, respectively.

^§^ Mann–Whitney test. Brazil, 2007.

**Figure 1:**
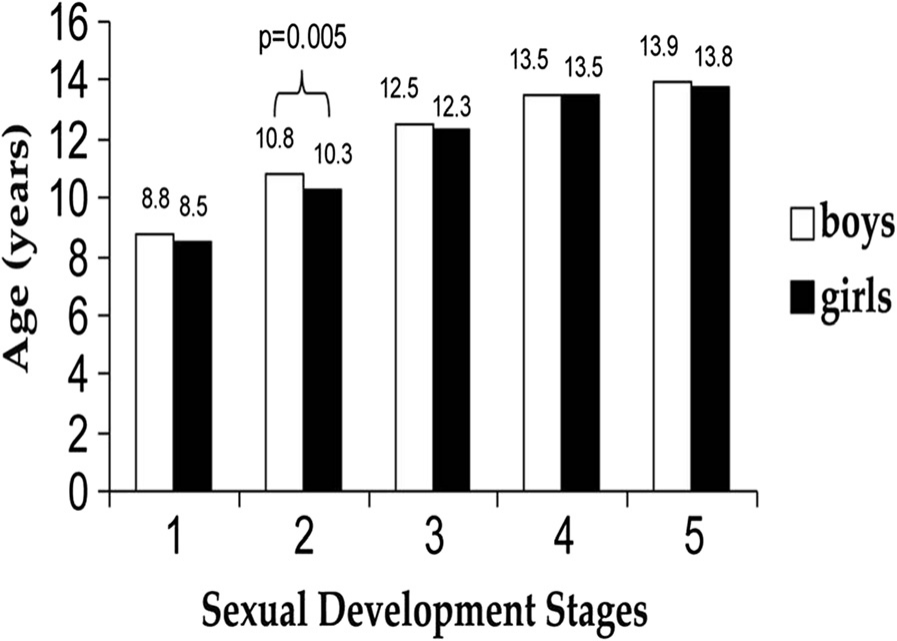
**Median ages in sexual development stages in boys and girls.** Brazil, 2007.

**Figure 2:**
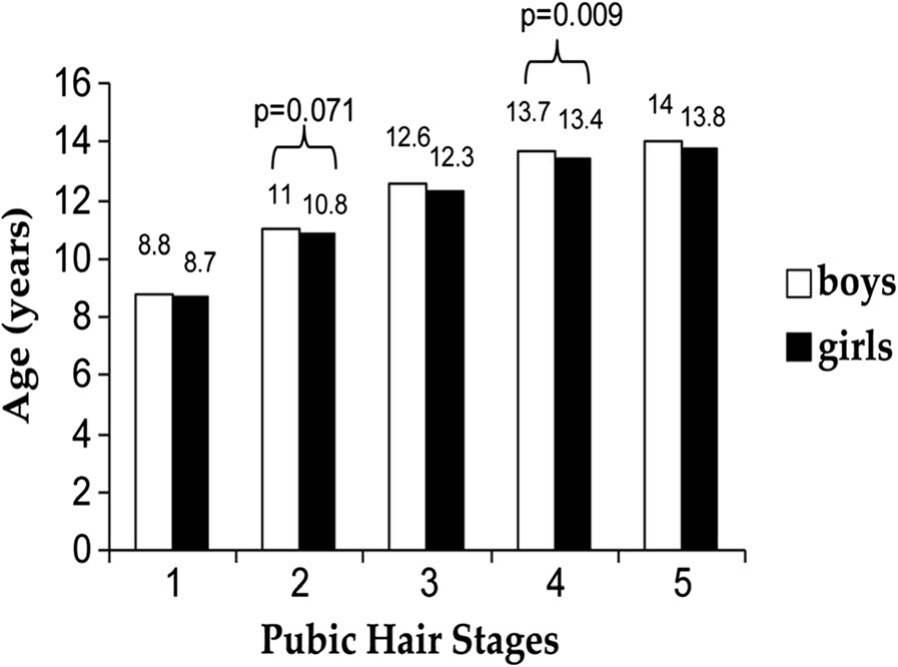
**Median ages in pubic hair stages in boys and girls.** Brazil, 2007.

Figures 1 and 2 show the median values of age for genital/breast development (Figure 1) and pubic hair (Figure 2) in boys and girls. A statistically significant difference between genders was found for stage 2 sexual development (p = 0.005), and stage 4 pubic hair (p = 0.009), and we can also highlight stage 2 pubic hair (p = 0.071). The median ages for the onset of puberty (stage 2 sexual development) in boys and girls in Florianopolis were 10.8 and 10.3 years, respectively.

## Discussion

Considering that the determination of age at the puberty beginning is suggested as an indicator to monitor growth, our study investigated whether excess weight is a modifying variable in the estimation of median ages for sexual maturity. As a main finding, we observed that excess weight is a relevant variable related to the determination of median ages for sexual maturity stages in boys and girls.

We reported in this study that in girls, all stages presented median ages lower than individuals with excess weight, which is the same tendency observed for pubic hair. Parent et al. [30] made a review of studies from various countries that determined the age for the onset of puberty in girls, identified through stage 2 breasts, according to Tanner criteria (1962) [20] the median age at that stage was 10.5 years. In boys, data points to the age of 10 years for the onset of puberty (identified through stage 2 genitalia development) [30]. In relation to stage 5, the literature shows that the development of gynecomastia and genitalia in boys continues until the age of 15 to 16 years [30].

Until recently, science had not associated human obesity directly with a mutant gene [2,31,32]. Researchers have identified a specific defect in two genes that produces a protein called leptin, a hormone that regulates body weight [33,34]. Studies with strains of hybrid and obese mice suggest that some individuals seem to be genetically pre-destined to becoming obese. The mutation of a gene named *obese* (*ob*) may affect the hormonal signals that regulate the metabolism, the storage of fat, and the appetite, which will lead to the accumulation of body fat [35]. The *ob* gene is activated normally in the adipose tissue, where it stimulates the production of leptin, which would regulate the signals pertaining to body fat. This protein, which modulates satiety, goes to the ventromedial nucleus of the hypothalamus, whose function is to control appetite and metabolism [36].

The levels of leptin found in obese children were higher than those of children with a normal Body Fat Index (BMI) [37]. The correlation between gender and the puberty stage were also related to the levels of leptin, which were higher in girls than in boys [37].

Studies have shown a dramatic increase in serum leptin in females who start puberty early, at 7 years of age, and that continues as puberty progresses, until at least 15 years of age [38]. In contrast, in boys, the levels of serum leptin seemed to increase temporarily, but decreased after Tanner stage 2 to pre-pubescent levels, and represented approximately one third of what was seen in girls at the final stage of puberty [38]. These alterations in serum levels of leptin are in parallel with and are the reflection of an increase in body fat during female puberty, but, even though present, they may not be so affected by the same phenomenon in boys.

It is already known that the increase in serum leptin levels happens approximately two years before the increase in LH and estradiol levels. This would be consistent with the hypothesis that more elevated levels of serum leptin could be one of the important factors that allow weight gain and the onset of puberty, instead of a result of hormonal increases in puberty itself [39].

Therefore, it is becoming clearer that leptin has direct effects on the secretion of gonadotropic hormones and establishes a connection between body fat and the time of puberty. Evidence suggests that a threshold level of leptin may be needed and that it is connected to the onset of puberty [39].

Some limitations of the study should be acknowledged. Firstly, causal interpretation on the association between excess weight and sexual maturity needs to be considered with caution, due to the observational nature and cross-sectional design of the study. Secondly, sexual maturity was self-assessed. This procedure in field research has been validated in studies with Brazilian adolescents, whose results have shown good correlation (r = ~0.80) between the measurements coming from self-evaluation and those made by a specialized professional [40,41]. On the other hand, there are some strengths of the study such as the representative large sample and the objective measure of weight and height by a well-qualified team.

## Conclusion

Excess weight is associated with early sexual maturity and that it should be taken into account when evaluating sexual maturity in children and adolescents.

## Consent

Written informed consent was obtained from the patient’s guardian/parent/next of kin for the publication of this report and any accompanying images.

## Competing interests

The authors declare that they have no competing interests.

## Authors’ contributions

All authors participated in the acquisition of data and revision of the manuscript. All authors determined the design, interpreted the data and drafted the manuscript. All authors read and gave final approval for the version submitted for publication.
